# Is chest X-ray screening for lung cancer in smokers cost-effective? Evidence from a population-based study in Italy

**DOI:** 10.1186/s12962-015-0041-0

**Published:** 2015-09-12

**Authors:** Paolo Pertile, Albino Poli, Lorenzo Dominioni, Nicola Rotolo, Elisa Nardecchia, Massimo Castiglioni, Massimo Paolucci, William Mantovani, Andrea Imperatori

**Affiliations:** Department of Economics, University of Verona, Via dell’Artigliere 19, 37129 Verona, Italy; Department of Public Health and Community Medicine, University of Verona, Verona, Italy; Center for Thoracic Surgery, University of Insubria, Ospedale di Circolo, Varese, Italy; Department of Radiology, Ospedale S. Antonio Abate, Gallarate, Italy; Department of Prevention, Public Health Trust, Trento, Italy

**Keywords:** Lung cancer, Chest X-ray screening, Cost-effectiveness, Cost-utility

## Abstract

**Background:**

After implementation of the PREDICA annual chest X-ray (CXR) screening program in smokers in the general practice setting of Varese-Italy a significant reduction in lung cancer-specific mortality (18 %) was observed. The objective of this study covering July 1997 through December 2006 was to estimate the cost-effectiveness of this intervention.

**Methods:**

We examined detailed information on lung cancer (LC) cases that occurred among smokers invited to be screened in the PREDICA study (Invitation-to-screening Group, n = 5815 subjects) to estimate costs and quality-adjusted life-years (QALYs) from LC diagnosis until death. The control group consisted of 156 screening-eligible smokers from the same area, uninvited and unscreened, who developed LC and were treated by usual care. We calculated the incremental net monetary benefit (INMB) by comparing LC management in screening participants (n = 1244 subjects) and in the Invitation-to-screening group versus control group.

**Results:**

The average number of QALYs since LC diagnosis was 1.7, 1.49 and 1.07, respectively, in screening participants, the invitation-to-screening group, and the control group. The average total cost (screening + management) per LC case was higher in screening participants (€17,516) and the Invitation-to-screening Group (€16,167) than in the control group (€15,503). Assuming a maximum willingness to pay of €30,000/QALY, we found that the intervention was cost-effective with high probability: 79 % for *screening participation* (screening participants vs. control group) and 95 % for *invitation*-*to*-*screening* (invitation-to-screening group vs. control group).

**Conclusions:**

Based on the PREDICA study, annual CXR screening of high-risk smokers in a general practice setting has high probability of being cost-effective with a maximum willingness to pay of €30,000/QALY.

## Background

Lung cancer (LC) is the most deadly malignancy, with an estimated 1,200,000 deaths each year worldwide [1]. More than 85 % of LC deaths are attributable to smoking cigarettes; therefore, tobacco control and smoking cessation are the interventions most commonly used to reduce LC morbidity and mortality [1, 2]. Because heavy smokers remain at high risk for many years, even after quitting [3], screening for LC is another intervention to consider. Screening aims to diagnose the disease at an early stage, when it is potentially curable with appropriate treatment protocols [4–7]. After lengthy methodological controversy over the efficacy of screening for LC, in recent years the results of observational screening with chest computed tomography (CT) in heavy smokers has shown a significant reduction of LC mortality [8]; with chest X-ray (CXR) screening the LC mortality reduction was less pronounced but still significant [9, 10]. After publication in 2011 of the National Lung Screening Trial (NLST) results, which showed a 20 % reduction in LC mortality with CT screening compared with CXR screening [11], major cancer organizations began to advocate for low-dose chest CT screening with the NLST criteria [12]. However, depending on the model and the assumptions applied, estimates of the cost-effectiveness of CT screening ranged widely, from $19,500 (USD) [13] to $2,322,700 [14] per quality-adjusted life-year (QALY) gained. The wide divergence of these estimates contributes to the current uncertainty about cost-effectiveness and affordability of CT screening in the general population setting [15–18]. While CT screening has been extensively analyzed, including its economic implications, the assessment of cost-effectiveness of CXR screening in smokers has received little attention. CXR as a LC screening tool is definitely less sensitive than chest CT [19], however it is more specific [20] and there are several clinical reasons why the cost-effectiveness of screening with chest radiograph may be worth investigating. With CXR screening there is not only a lower probability of false positive tests, but also less LC overdiagnosis and lower radiation doses than with CT screening [21, 22]. Moreover, from an economic perspective CXR is a relatively inexpensive and broadly accessible exam, two highly favorable features in a screening tool. Despite this, we are only aware of one cost-effectiveness analysis of CXR screening, based on the model developed by Caro et al. [23], where the estimated cost per discounted life-years gained ranged from $19,874 (USD) to $59,621 (USD). Before that, Strauss [24] made only a “crude analysis” (p. 768), leading to an estimate of $7900 per life-year saved. It was previously reported that a population-based CXR screening program which targeted smokers (PREDICA) decreased LC mortality by 18 % [9], and we made an initial estimate of the total incremental cost of screening [25]. In this paper we use the data from the PREDICA cohort [26] to investigate the cost-utility of CXR screening for LC. Our aim is to evaluate the cost-utility of the screening program in the total *invitation*-*to*-*screen PREDICA cohort*, including self-selected participants as well as individuals who were invited to the screening but decided not to participate. With this “intention-to-screen approach”, the analysis of cost-effectiveness overcomes the well-known bias of participants’ self-selection [27] and allows estimates of the screening program’s overall impact on the target population.

## Methods

### Overview of the PREDICA study

In 1997 we began a prospective observational study, the PREDICA study (ISRCTN90639073), in a clearly defined population-based cohort of smokers. The cohort consisted of all smokers of >10 pack-years, aged 45–75 years, resident in 50 communities of the Province of Varese-Italy, who were screening-eligible (n = 5815 subjects). These subjects were invited by their National Health Service (NHS) general practitioner to receive an annual LC screening with CXR for 4 years, according to an established protocol [26]. The PREDICA cohort was observed from July 1, 1997 until the end of the study (December 31, 2010), a total of 13.5 years. The cohort’s demographic characteristics, adherence to screening, LC detection results, LC survival rate, and LC mortality have been published [9, 26]. Because some information relevant to the economic analysis was missing after 2006, the observational interval used in this study ends with December 31, 2006. For the period until the end of 2006, clinical data were gathered, which allow to assign each patient to one of the LC stages associated with a specific value of the Quality of Life, presented later under “*Utilities”*. Moreover, the main events related to substantial resource use were also recorded, including surgery, radiotherapy, chemotherapy, follow-up exams.

### Defining the invitation-to-screening group and screening participants

In the present study of cost-effectiveness of CXR screening, the PREDICA cohort of 5,815 subjects is referred to as the *Invitation*-*to*-*screening Group*. Of the subjects invited to the screening program, 1244 (21 %) agreed to participate and are referred to as the *Screening Participants*.

### Defining lung cancer patient/lung cancer death

In the Invitation-to-screening Group we defined LC patients as those who presented with a new clinical or pathological diagnosis of LC—after excluding screening-ineligible candidates (i.e., subjects unfit for surgery or with diagnosed or suspected LC as of July 1997). Through linkage with the Varese Mortality Registry and the Lombardy Health Registry of residents we identified LC deaths (codes 162.2–162.9 of the International Classification of Diseases, Edition IX) that occurred in the Invitation-to-screening Group during the study period. Information on date and cause of death were obtained from the Registry death certificates. Cause of death was definitively attributed to LC after review by the mortality review committee, as previously described [26]. The characteristics of the LC cases reported for the Screening Participants, the Invitation-to-screening Group, and the Control Group are summarized in Table 1.

**Table 1:** Characteristics of lung cancer (LC) cases diagnosed between July 1, 1997 and December 31, 2006 in screening participants, invitation-to-screening group and control group [26]

Variable	Screening participants (67 LC cases)	Invitation-to-screening group (245 LC cases)	Control group (156 LC cases)
Age, median (IQR)	66 (60–72)	68 (60–73)	68 (62–73)
Gender M/F	60/7	221/24	144/12
Smoking habit, n (%)^a,b^			
Ex smokers	16 (24)	60 (25)	77 (49)
Smokers	50 (76)	180 (75)	79 (51)
LC stage at diagnosis, n (%)			
I	21 (32)	49 (21)	23 (15)
II	3 (5)	13 (6)	9 (6)
III/IV	41 (63)	166 (73)	122 (79)
Indeterminate/not available	2	17	2
LC cases with censored follow-up*, n (%)	21 (31 %)	57 (23 %)	12 (8 %)
LC follow-up months*, median (IQR)	14.03 (30.82)	11.89 (22.54)	8.20 (15.82)

*IQR* interquartile range, *CI* confidence interval

* Expressed as months from LC diagnosis until death, or until the end of observational period (December 31, 2006)

^a^Not available in 1 screening participant

^b^Not available in 5 subjects of invitation-to-screening group

### Timeline of economic evaluation of screening and follow-up of lung cancer cases

For the purposes of the economic analysis, the LC cases were followed until death or December 31, 2006. For patients alive at the end of the observational period, we have censored observations. The different proportions of these cases in the different groups (see Table 1)—due partly to the different recruitment periods—implies a potential bias in the analysis from underestimation of both costs and QALYs for these patients. The way in which we addressed this potential source of bias is discussed below under “*Utilities”* and “*Costs”.*

### Control group

A Control Group consisting of uninvited and unscreened LC patients, prospectively followed-up long term, was identified and used as comparator for LC cases found in the Invitation-to-screening Group. We accessed the database of all LC cases (n = 243) diagnosed during calendar year 2000 among the 350,000 residents of the Varese district. The year 2000 LC cases were chosen because (1) they were nearly contemporary with the LC cases of the Invitation-to-screening Group, (2) they came from the same geographical area, and (3) their demographic and clinico-pathological data were available and published [28]. Of the 243 LC patients diagnosed in 2000, 156 subjects met the screening criteria as of July 1997 (birth-year cohort, smoking history, residence in the district of the cohort, uninvited to screening and unscreened) and constituted the Control Group. Follow-up of these 156 LC cases was obtained through linkage with the Varese Epidemiology Observatory [29].

### Management of lung cancer patients

The LC cases diagnosed in the Invitation-to-screening Group—regardless of the detection modality by screening or outside screening—and the LC cases diagnosed in the Control Group both received NHS’s usual care and treatment, with management centralized in the Varese University Hospital. The diagnostic procedures for follow-up and diagnosis of individuals with suspect LC have been described previously [26]. For patients who refused biopsy or treatment and for candidates for supportive care only, the LC diagnosis was clinico-radiologic. Treatment of ascertained LC cases—surgery, chemotherapy (induction and/or adjuvant), radiotherapy, palliation—was effected following international criteria [28], based on histologic subtype (non-small cell lung cancer, small cell lung cancer) and stage (6th ed. of TNM Classification of Malignant Tumors) [30].

### Economic evaluation

We assessed LC screening program cost-utility using the public health system perspective: *utility* was measured through QALYs. We estimated the incremental cost-utility of LC screening through two comparisons. The obvious comparison was between screening participants and the control group; the other comparison was between the Invitation-to-screening group and the control group. The rationale for the latter comparison was to avert the self-selection bias of participants [27] and to explore the overall impact of invitation to screening (greater attention to early symptoms of LC among all invitees and among the general practitioners who accepted to recruit smokers for the PREDICA study).

### Utilities

To assign utilities, for each patient we considered the duration of the following phases of LC clinical course: diagnosis, chemotherapy, post-operative, free of disease, progression, terminal phase. For each phase we obtained from the literature an estimate of the corresponding quality of life (QoL), specifically for non-small cell lung cancer (NSCLC) and for small cell lung cancer (SCLC). Phases and corresponding weights are reported in Table 2 for the baseline scenario [31] and for an alternative scenario based on the results of a meta-analysis [32] used for sensitivity analysis (columns 3 and 4).

**Table 2:** Phases of lung cancer clinical course and Quality of Life (QoL)

Phase of clinical course of lung cancer	QoL Index			
	NSCLC (baseline)	SCLC (baseline)	NSCLC (alternative scenario)^a^	SCLC (alternative scenario)^a^
1. Period of diagnosis	0.88^d^	0.95^d^	0.825^d^	0.605^d^
2. Chemotherapy	0.82^a^	0.83^a^	0.573^c^	0.353^c^
3. Postoperative	0.80^b^	0.80^d^	0.825^c^	0.605^c^
4. Free of disease	0.88^a^	0.95^a^	0.825^c^	0.605^c^
5. Disease progression	0.69^a^	0.31^a^	0.573^c^	0.353^c^
6. Terminal phase	0.60^b^	0.31^d^	0.573^c^	0.353^c^

*NSCLC* non-small cell lung cancer,* SCLC* small cell lung cancer

^a^Alternative scenario described in “Methods”; Earle et al. [33]

^b^Manser et al. [31]

^c^Sturza [32]

^d^Where specific information on the QoL index could not be retrieved from the literature, we made the following assumptions: In column 2, for the “postoperative” phase we assumed the index for SCLC to be the same as for NSCLC, Line 1 is the same as line 3, In column 2, for the “terminal” phase we assume the index to be the same as for the “progression”

A correction of QoL for false positive screens related to anxiety (QoL index = 0.88 for 3 months) was made, according to Earle et al. [33].

After reviewing the literature on LC screening with CT, Black et al. could not draw any conclusions on whether the intervention was cost-effective [34]; it had been observed that the variety of modelling assumptions made in different economic evaluations is the main reason for the widely divergent estimates of cost-effectiveness of CT screening for LC [35]. In the present study we relied on modelling as little as possible, utilizing instead the wealth of data gathered from long-term observation of the Invitation-to-screening Group. In particular, to correct for potential bias from the varying proportions of censored survivors in the different groups, while bearing in mind the issue of ambiguity related to modelling assumptions that Black et al. [34] highlight, we used information from non-censored individuals to correct the value of utility assigned to censored observations. In this process we assigned to each of these survivors a number of QALYs equal to the median of a subset of cases with the longest follow-up (those recruited in 1997 and 1998 in the PREDICA study), conditional on the length of survival at the end of the study and whether the individual was screen-detected. The added number of QALYs was the excess, if any, between this conditional median and the actual number of QALYs through end of follow-up.

A frequent omission in LC screening economic evaluations is the lead-time bias correction [34]. In our study we assumed a lead-time bias of 9 months, based on stage distribution of screen-detected LCs in the PREDICA study [26]; then we subtracted from the utility of screen-detected LC cases the minimum between 9/12 of QALY and the actual number of QALYs. This is a conservative assumption because it leads to the attribution of zero QALYs to some screen-detected patients even though they survive for a number of months. Underlying this is another conservative assumption, i.e. that the corresponding months would be spent in perfect health (QoL index = 1). Utility values were discounted using a yearly discount rate of 3.5 % [36].

### Costs

Costs of LC cases were linked to three main activities: administrative work to run the screening program, screening exams, and health care services. Data on administrative costs of screening were extracted from the balance-sheet of the charity that managed the screening program; voluntary work was evaluated using market-equivalent wages. CXR screening exam costs included the cost of follow-up exams to ascertain suspicious or false positive CXR screens and the cost of time spent for quality assurance and program supervision. For the latter, we estimated a workload equivalent to 20 % of a full-time consultant physician for the duration of active screening. Unitary costs for health care treatment and follow-up were estimated from the corresponding 2012 regional tariffs, which are reported in Appendix (Table 5). These may differ slightly from National tariffs, although in several cases they are the same across Italy.

Because utilities were adjusted to account for censoring, the adjustment was also done for costs. We used the same approach as that described above for utilities, i.e. we assigned the median cost, conditional on survival at the end of follow-up and whether the case was screen-detected.

All values were expressed in 2012 Euros using the Italian National Institute of Statistics [37] health care price index. The discount rate used for utilities (3.5 %) was applied to costs.

### Cost-utility analysis

The cost-effectiveness of a new intervention is usually assessed by comparing the Incremental cost-effectiveness ratio (ICER) to a threshold value of the maximum Willingness to Pay (WTP) per unit of effectiveness:

$$\frac{\Delta C}{ \Delta E} < \lambda ,$$

where $\Delta C$ and $\Delta E$ are the difference, respectively, in costs and effectiveness of the comparator, and $\lambda$ is the WTP. The inequality in the above equation can be equivalently expressed in linear form as:

$$\lambda \,{ \cdot }\, \Delta E - \Delta C > 0.$$

The left side of the equation is the Incremental Net Monetary Benefit (INMB). The latter was proposed as an alternative to ICER [38] mainly because of the advantages it provides in statistical analysis; it is also widely used currently. The INMB measures the incremental net benefit of the new intervention in monetary units. In our case, the INMB is based on a measure of utility (QALYs) and can be calculated as a weighted average across health states of individuals:

$$INMB = p_{LC}\, { \cdot }\,INMB_{LC} + p_{fp} \,{ \cdot }\,INMB_{fp} + \left( {1 - p_{LC} - p_{fp} } \right)\,{ \cdot }\,INMB_{tn}$$

where, $p$ denotes probability and subscripts refer to the following states: lung cancer ($LC$), false positive ($fp$) and true negative ($tn$). $INMB_{LC}$ is calculated using data on costs and utilities that are available for both the *invitation*-*to*-*screening group* and the *control group*. $INMB_{fp}$ is estimated using data from the *screening participants* and it is based again on a conservative assumption, i.e. that only screening may lead to a false positive response. $INMB_{fp}$ is a negative number that accounts for both additional costs and anxiety-related QoL implications of a false positive screen. Finally, $INMB_{tn}$ is also a negative number because of the costs (administration and screening) assessed even if the individual remains healthy. We assume no difference in utility among the groups compared for the true negative.

In addition to the well-known advantages of INMB over Incremental Cost-Effectiveness Ratio (ICER) for dealing with sampling variance [39], the former approach also allows us to study the sensitivity of our results to the probability of diagnosed LC and false positive test. This is crucial for three reasons: (1) the results tend to be very sensitive to these probabilities; (2) the number of LC cases that we observed among participants (67) is relatively small, and (3) the statistical error for the estimated probability of diagnosed LC may be comparatively large. A sensitivity exercise on these probabilities may help assess the possibility of extending our results to contexts in which LC risk is higher or lower.

We made two estimates of the INMB. First, we compared Screening Participants vs. Control Group, then Intervention-to-screening Group vs. Control Group. We interpret these results as the cost-utility of *screening*-*participation* and *invitation*-*to*-*screening,* respectively.

### Sensitivity analysis

For baseline analysis, we used the frequency of LC cases in Screening Participants and in Intervention-to-screening Group, after excluding 2 cases of overdiagnosis (67/1244 = 0.054 and 243/5815 = 0.042), to estimate $p_{LC}$. Similarly, $p_{fp}$ equals the ratio of the number of individuals with a false positive test to the number of participants (190/1244 = 0.153). Sensitivity analysis was performed by replacing the baseline probability with the extremes of the 95 % confidence interval (CI) of the estimated probability of LC. The other parameters we let vary in the sensitivity analysis were QoL weights (see Table 2) and cost levels. For costs, we investigated the impact of an arbitrary 20 % increase in all costs, which may inform about the possibility of generalizing our results to other health care systems. We primarily used two values of the WTP that are often referred to in the literature: € 30,000/QALY and €50,000/QALY. A general analysis of the impact of the WTP was provided by estimating the cost-effectiveness acceptability curve (CEAC).

## Results

Mean values for QALYs and costs of LC cases are shown in Table 3. Overall, the mean total cost per LC case was higher in Screening Participants (€17,516) than in the control group (€15,503). The individual component of the costs related to organization and administration of the screening program (€307) was obtained by dividing the total cost by the number of participants. This component is the same for LC cases (Table 3) and true negative individuals in Screening Participants. The LC management costs were also higher in Screening Participants (€17,149) than in the control group (€15,503).

**Table 3:** Mean costs and QALYs per lung cancer (LC) case in screening participants, invitation-to-screening group and control group

	Screening participants	Invitation-to-screening group	Control group
Mean costs per LC case			
Screening organization and administration	307 (0)	84 (0)	–
CXR screening	60 (44)	16 (35)	–
LC management	17,149 (4783)	16,067 (4948)	15,503 (4518)
Mean total cost per LC case	17,516 (4755)	16,167 (4729)	15,503 (4521)
Mean QALYs (baseline scenario)	1.70 (2.1)	1.49 (1.9)	1.07 (1.7)

All amounts are in Euros 2012. Standard deviation in parenthesis. Values are rounded to the nearest integer

Looking at the comparison between the Invitation-to-screening Group and the Control Group, the incremental total cost (€16,167–15,503) was €664. Costs related to screening activities were lower for the Invitation-to-screening Group (€100) than for screening participants. This is because the Invitation-to-screening group includes non-participants for whom screening costs were imputed to Screening Participants (see above). Finally, LC management cost was higher, on average, in the Invitation-to-screening Group (€16,067) than in the control group (€15,503) (Table 3).

The average (discounted) number of QALYs since diagnosis was 1.70, 1.49, and 1.07, respectively, for screening participants, the invitation-to-screening group, and the control group. Accordingly, on average Screening Participants gained 0.63 QALYs and the Invitation-to-screening Group gained 0.42 QALYs, relative to the Control Group. The combination of these results with costs and utilities of true negative and false positive individuals leads to an estimate for the individual level INMB of €368 for *screening participation* and €365 for *invitation*-*to*-*screening*, with a WTP of €30,000 per QALY (Table 4). With a WTP of €50,000 per QALY, the estimated INMB increases, respectively, to €959 for *screening participation* and €692 for *invitation*-*to*-*screening*. Table 4 also shows the probability that, taking sampling variance into account, the intervention is cost-effective for the two levels of WTP considered, under the main and the sensitivity scenarios. In particular, it is worth noting that a 20 % increase in unitary costs does not significantly change the results.

**Table 4:** Sensitivity analysis testing the probability that *screening participation* or *invitation*-*to*-*screening* is cost-effective

	INMB		Probability cost-effective	
	WTP (€ per QALY) 30,000	WTP (€ per QALY) 50,000	WTP (€ per QALY) 30,000	WTP (€ per QALY) 50,000
Screening participation (group P vs. group C)				
Base-case	368	959	0.79	0.89
High prob of LC (*p*_*LC*_ = 6.79 %)	609	1376	0.86	0.92
Low prob of LC (*p*_*LC*_ = 4.2 %)	160	600	0.67	0.84
Alternative QoL	134	568	0.63	0.80
Higher costs	265	856	0.72	0.87
Invitation-to-screening (group I vs. group C)				
Base-case	365	692	0.95	0.97
High prob of LC (*p*_*LC*_ = 4.73 %)	438	816	0.96	0.97
Low prob of LC (*p*_*LC*_ = 3.68 %)	311	601	0.94	0.97
Alternative QoL	260	519	0.90	0.93
Higher costs	339	667	0.94	0.96

*INMB* incremental net monetary benefit, *WTP* willingness to pay, *LC* lung cancer

**Fig. 1:**
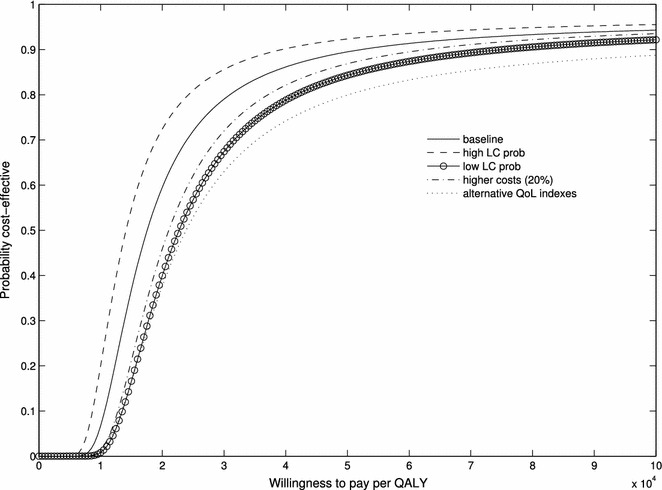
Cost-effectiveness acceptability curve for *screening participation (screening participants* vs. *control group)* in the baseline and sensitivity scenarios (see text, “Sensitivity analysis”)

**Fig. 2:**
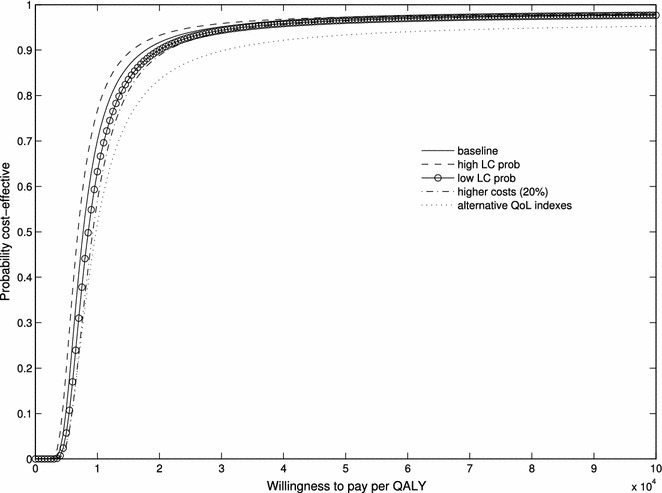
Cost-effectiveness acceptability curve for *invitation*-*to*-*screening (invitation*-*to*-*screening group* vs. *control group)* in the baseline and sensitivity scenarios (see text, “Sensitivity analysis”)

The CEAC provides a more general picture of the relationship between the level of WTP and the probability that screening is cost-effective. Figures 1 and 2 show the CEAC for the baseline and sensitivity scenarios. Referring once more to the values of WTP per QALY of €30,000 and €50,000, the probability that *screening participation* is cost-effective is, respectively, 0.79 and 0.9 in the baseline scenario. The corresponding probabilities for *invitation*-*to*-*screening* are 0.95 and 0.97. Table 4 summarizes the results of the sensitivity analysis.

## Discussion

Based on long-term observation of the population-based PREDICA study of CXR screening in smokers [26], our assessment of cost-effectiveness of CXR screening for LC indicates an expected INMB (WTP: €30,000 per QALY) of €368 for the comparison between Screening Participants and Control Group in the baseline analysis. This finding implies a 0.79 probability that *screening participation* is cost-effective. Notably, in addition to screening-related costs, participants showed higher LC management costs due to more frequent surgical procedures performed as a result of early LC detection. Moreover, compared with the Control Group, the Screening Participants had markedly improved 5-year survival (30.5 vs. 13.0 %) [26] and longer median follow-up (Table 1), which added cost to LC management. Because we observed only 67 LC cases among Screening Participants (n = 1244), despite the large number of individuals (n = 5815) invited to screening, the variance around the central estimate of the INMB is wide. This confirms that the assessment of a screening program cost-effectiveness requires substantial investment of resources and prolonged observation.

Our study has limitations. First, it is not a randomized study and therefore is subject to the selection bias of screening volunteers, as previously noted [27]. Second, in our study the proportion of censored observations was different across groups. We addressed this potential bias by adjusting utilities and costs of censored observations using the wealth of information available on non-censored patients.

A strength of our study is the Control Group consisting of uninvited and unscreened LC patients, with 92 % complete follow-up. The Control Group LCs were nearly contemporary to those of the Invitation-to-screening Group and were gathered from the same geographical area. Another strength of the study is that information was gathered on LC cases that occurred in individuals who were invited to the screening program and decided not to participate. Thus, we were able to assess the overall cost-utility of *invitation*-*to*-*screening* by comparing the Invitation-to-screening Group with the Control Group. This comparison yielded an expected value of individual INMB of €365, indicating that *invitation*-*to*-*screening* is cost-effective with a probability of 0.95. The small difference with the central estimate of INMB obtained from the previous comparison (€368 vs. €365) is due to the fact that invited subjects who did not participate had LC costs similar to the Control Group, but achieved more QALYs.

Clearly, our estimates may be affected by local context (costs, incidence of LC, WTP); however, our probabilistic sensitivity analysis showed limited impact of changes in these variables on results.

The estimated probability that the intervention is cost-effective is higher for *invitation*-*to*-*screening* than for *screening participation,* despite the very close central estimates for the INMB. This finding is related to the variance of the sample mean, which is greater when Screening Participants (a subset of the invitation-to-screening group) are compared with the Control Group (67 LC cases vs. 156). Moreover, screening invitation per se, regardless of participation, might have an impact on LC management. The impact of invitation and its economic implications are potentially of great interest and worth exploring further in different contexts. There may also be relevant policy implications. Because the number of invited individuals typically exceeds the number of participants in screening programs, the total net benefit of the program (individual INMB times the population size) should also take into account any impact on the invited population, in addition to the effect on participants.

Results from the PREDICA study suggest that the total net benefits of a LC screening program may be greater than expected based on the analysis of screened individuals only. Implementation of the PREDICA annual CXR screening program at population level in smokers of the Varese area was associated with an 18 % reduction of LC-specific mortality [9]. Because our study shows that CXR screening of high-risk smokers can be “good value for the money”, the findings may help decision-makers allocate more efficiently the available health care resources. The NLST trial showed that screening for LC with CT reduced LC mortality by 20 % compared with CXR screening [11]. Based on this finding, leading cancer organizations currently recommend LC screening with LC low-dose CT [12, 40]. However, low-dose CT screening is an expensive technique not readily available in many parts of the world. Moreover, low-dose CT screening is associated with a relevant rate of overdiagnosis and of false positive findings [11, 41]. It has been emphasized that if early detection of LC provides a benefit, it does so regardless of whether the detection is made by CT or CXR screening; however, the feasibility of the detection method applied to a large number of subjects as a public health measure is essential [42]. Chest radiography is a simple exam with modest radiation exposure. It is widely available, relatively inexpensive, and non-invasive. Additionally, CXR screening causes negligible overdiagnosis of LC [21] and a low rate of false-positive results [22, 26].

In conclusion, based on the PREDICA study [9, 26], annual CXR screening of high-risk smokers in the general practice setting is a feasible and affordable intervention that has high probability of being cost-effective. This finding may have important policy implications in terms of resource allocation in health care systems. However, before recommending annual screening of heavy smokers by CXR, the results obtained in the present study need to be validated by other similar studies in different geographical areas and different health systems.

## Appendix

See Table 5.

**Table 5:** Cost of health care services for diagnosis, staging, treatment and follow-up of lung cancer

Cost components	Unitary cost (EURO 2012)	Source
Cyto-/histologic confirmation of NSCLC/SCLC		
Bronchoscopy with biopsy	184.74	Tariff 2012 BRL
Cytology of sputum/pleural fluid	27.45	Tariff 2012 BRL
CT-guided fine needle aspirate and cytology	160.99	Tariff 2012 BRL
Day hospital stay	624.00	Tariff 2012 BRL
Total	997.18	
Evaluation of stage I–IV LC		
Blood cells count, chemistry, markers, gases	117.25	Tariff 2012 BRL
EKG	11.60	Tariff 2012 BRL
CXR exam, dual projection	17.40	Tariff 2012 BRL
Chest CT without and with contrast	164.67	Tariff 2012 BRL
Abdomen CT without and with contrast	168.37	Tariff 2012 BRL
Head CT without and with contrast	159.93	Tariff 2012 BRL
Bone scintigraphy	111.90	Tariff 2012 BRL
Spirometry	23.75	Tariff 2012 BRL
Hospital stay (average 7 days)	4160.00	Tariff 2012 BRL
Total	4934.87	
Evaluation of indeterminate stage LC		
Blood cells count, chemistry, markers, gases	117.25	Tariff 2012 BRL
EKG	11.60	Tariff 2012 BRL
CXR exam, dual projection	17.40	Tariff 2012 BRL
Chest CT without and with contrast	164.67	Tariff 2012 BRL
Abdomen CT without and with contrast	168.37	Tariff 2012 BRL
Head CT without and with contrast	159.93	Tariff 2012 BRL
Hospital stay (average 4 days)	2496.00	Tariff 2012 BRL
Total	3135.22	
Operating room occupancy (average 180 min.)	3000.00	Varese Hospital Admin.
Operating room materials	1500.00	Varese Hospital Admin.
Hospital stay in surgical unit (average 12 days)	6600.00	Varese Hospital Admin.
Total	11,100.00	
Chemotherapy for LC stage III		
6 Cycles (Cisplatin + Gemcitabine)	8989.62	Oncology Unit Varese Hospital
Chemotherapy for LC stage IV		
3 Cycles (Cisplatin + Gemcitabine)	4494.81	Oncology Unit Varese Hospital
Palliative radiotherapy	4934.55	Radiotherapy Unit Varese Hosp.
Radical radiotherapy (50–60 Gy)	7849.46	Radiotherapy Unit Varese Hosp
Chemo-Radiotherapy combined	10,091.03	Onc./Radiot.Units Varese Hosp.
Supportive/palliative therapy	2759.76	Tariff 2012 BRL
Terminal phase care (1 month)	1800.00	Tariff 2012 BRL
Follow-up: first year		
3 Physical exams	74.10	Tariff 2012 BRL
2 CXR exams, dual projection	34.80	Tariff 2012 BRL
2 Blood cells counts, chemistry, markers, gases	234.50	Tariff 2012 BRL
1 Chest CT exam without and with contrast	164.67	Tariff 2012 BRL
Total	508.07	
Follow-up: second year		
2 Physical exams	49.40	Tariff 2012 BRL
1 CXR exam, dual projection	17.40	Tariff 2012 BRL
2 Blood cells counts, chemistry, markers, gases	234.50	Tariff 2012 BRL
1 Chest CT exam without and with contrast	164.67	Tariff 2012 BRL
Total	465.97	
Follow-up: third and subsequent years		
2 Physical exams	49.40	Tariff 2012 BRL
1 Blood cells count, chemistry, markers, gases	117.25	Tariff 2012 BRL
1 Chest CT exam without and with contrast	164.67	Tariff 2012 BRL
Total	331.32	

Source of information about cost is indicated

*NSCLC* non-small cell lung cancer, *SCLC* small cell lung cancer, *BRL* Bulletin of Region Lombardy, *CT* computed tomography, *CXR* chest X-ray, *EKG* electrocardiogram, *LC* lung cancer

## Abbreviations

CEAC: cost-effectiveness acceptability curve
CT: computed tomography
CXR: chest X-ray
*fp*: false positive
ICER: incremental cost-effectiveness ratio
INMB: incremental net monetary benefit
LC: lung cancer
NHS: National Health System
NLST: National Lung Screening Trial
NSCLC: non-small cell lung cancer
QALY: quality-adjusted life year
QoL: Quality of Life
*tn*: true negative
WTP: willingness to pay
